# RPA-CRISPR-Cas13a-assisted detection method of transmissible gastroenteritis virus

**DOI:** 10.3389/fvets.2024.1428591

**Published:** 2024-07-02

**Authors:** Haoyu Wang, Zhimeng Cheng, Ran Luo, Qiyue Yang, Yongping Zeng, Yijun Yang, Yuankun Chen, Wenting Li, Xiao Liu

**Affiliations:** ^1^Southwest University, College of Veterinary Medicine, Chongqing, China; ^2^Division of Biliary Tract Surgery, Department of General Surgery, West China Hospital, Sichuan University, Chengdu, Sichuan, China; ^3^Research Center for Biliary Diseases, West China Hospital, Sichuan University, Chengdu, Sichuan, China; ^4^Department of Infectious and Tropical Diseases, The Second Affiliated Hospital of Hainan Medical University, Haikou, China; ^5^Department of Infectious Diseases, The First Affiliated Hospital of Anhui Medical University, Hefei, China; ^6^State Key Laboratory of Silkworm Genome Biology, Chongqing, China

**Keywords:** porcine transmissible gastroenteritis virus (TGEV), recombinase polymerase amplification (RPA), CRISPR-Cas13a, detection, isothermal amplification

## Abstract

**Background and aim:**

Transmissible gastroenteritis virus (TGEV) is a highly contagious gastrointestinal virus that causes diarrhea, vomiting, anorexia, dehydration, and weight loss in piglets. In clinical practice, it often occurs in mixed infections with other pathogens, and is therefore difficult to diagnose and prevent. It mainly harms piglets of about 2 weeks old, causing huge losses on farms. The clinical confirmation of TGEV usually requires a laboratory diagnosis, but traditional PCR and immunofluorescence assays have some limitations. Moreover, most farms in China are ill-equipped to accurately diagnose the disease. Therefore, a new detection method with high sensitivity and specificity and less dependence on instrumentation is required.

**Methods:**

We used recombinase polymerase amplification (RPA), combined with the nuclease characteristics of the activated Cas13a protein to establish a visual CRISPR-Cas13a-assisted detection method for TGEV by adding a reporter RNA with fluorescent and quenching moieties to the system.

**Result:**

We selected the optimal RPA primer and best CRISPR RNA (crRNA). The reaction system was optimized and its repeatability, specificity, and sensitivity verified. The TGEV detection system did not cross-react with other common diarrhea viruses, and its detection limit was 10^1^ copies, which is similar with the sensitivity of qPCR. We successfully established an RPA–CRISPR-Cas13a-assisted detection method, and used this detection system to analyze 123 pig blood samples. qPCR was used as the gold standard method. The sensitivity, specificity, positive coincidence rate, and negative coincidence rate of the new method were 100, 98.93, 96.66, and 100%, respectively.

## 1 Introduction

Transmissible gastroenteritis virus (TGEV) is a member of the genus *Coronavirus* in the family *Coronaviridae*, and is an α-type coronavirus (1). TGEV causes a highly contagious intestinal disease in pigs, and is one of the important causes of piglet death. Therefore, it has brought huge losses to China’s pig-breeding industry (2, 3). TGEV has been prevalent in many regions of China since the first outbreak in 1956. At present, the inactivated vaccine against TGEV does not induce immune components in the milk of pregnant sows, which therefore confers no protection from infection on piglets. However, the attenuated vaccine often poses a risk of increased toxicity and requires multiple rounds of immunization. Therefore, the rapid and accurate diagnosis of TGEV infection is extremely important for the prevention and treatment of the disease (4, 5).

Because the isolation of TGEV is complicated in clinical practice and it is often present as co-infections with other viruses, such as Porcine epidemic diarrhea virus (PEDV) (6), it cannot be detected or diagnosed quickly. Moreover, diagnostic reverse transcription (RT)–PCR requires the relevant instrumentation and technically trained personnel in clinical practice. The equipment requirements are high, but the conditions on most farms in China are limited, so the diagnosis of TGEV infection is difficult. Recombinase polymerase amplification (RPA) is a new technology that can replace PCR detection (7). It uses recombinase, a strand displacement enzyme and single-stranded binding protein, to achieve the specific binding of primers and templates at room temperature. RPA can amplify the target sequence at 37°C, taking only 5–20 min, greatly reducing the time required for diagnosis. It also has little requirement for instrumentation, is time efficient, has high specificity and sensitivity, and can be used in environments in which PCR cannot (8).

The clustered regularly interspaced short palindromic repeats (CRISPR) technique was first developed in Japan in 1987 by scientists Ishino et al., and named by Mojica, Jansen (9–11). CRISPR-associated proteins form part of the immune system of prokaryotes, which captures integrated foreign sequences and guides immune function by RNA interference after identifying foreign sequences (12). In recent years, CRISPR has been used as a new type of nucleic acid detection technology, with high sensitivity and specificity, drawing the increasing attention of researchers and with broad applications in many disciplines. Among these proteins, Cas13a acts on RNA in the CRISPR system. Unlike Cas9 and Cas12a, it has an RNase function under the guidance of CRISPR RNA (crRNA). *Leptotrichia wadei* Cas13a (LwaCas13a) has no restriction on its protospacer flanking site (PFS) (13), making it extremely flexible when used as a nucleic acid detection tool. After activation, the Cas13a protein nonspecifically cleaves any surrounding single-stranded RNA (ssRNA) (14). Based on this feature, specific RNA fluorescent reporter probes can be designed to allow CRISPR-Cas13a-assisted nucleic acid detection. Therefore, the combination of RPA and CRISPR-Cas13a provides a more convenient and less instrument-dependent new option for the diagnosis of TGEV.

## 2 Materials and methods

### 2.1 Construction of positive plasmid

We selected the sequence fragment of TGEV-CHN-SC strain (GenBank ID: ON016092.1) from southwest China from the National Center for Biotechnology Information (NCBI), and inserted it into the pUC57 plasmid, using the N-region sequence (Site: 26888 to 28,036) (1,149 bp) of TGEV as the template upon which to construct the positive plasmid pUC57–TGEV. The 1ul (1 ng) pUC57–TGEV positive plasmid was transformed into DH5α competent *Escherichia coli* (Shanghai Weidi Biotechnology Co., Ltd.), then screened and cultured competent *Escherichia coli* on Luria–Bertani (LB) agar containing ampicillin. The colonies were selected and inoculated into LB liquid, and cultured overnight at 37°C and 180 rpm. Centrifuge the broth at 8000 rpm for 10 min. We used the plasmid mini kit (Tiangen Biotechnology Co., Ltd.) to extract the plasmid from the cells, and stored it at −80°C.

### 2.2 Primers, crRNA, and reporter synthesis

**Figure 1:**
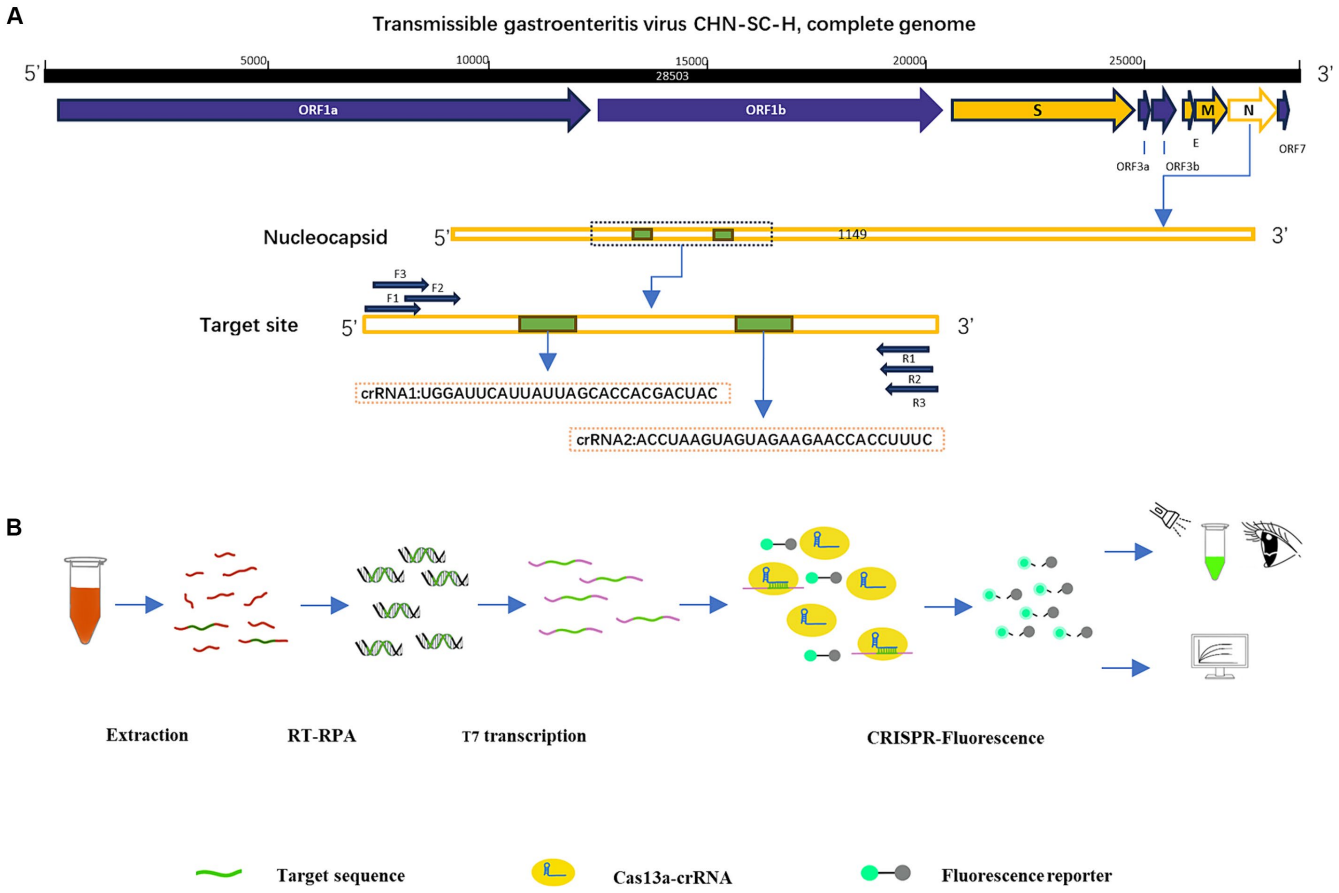
Construction of RPA–CRISPR-Cas13a detection system. **(A)** Design of crRNA and primers in the N region of TGEV. **(B)** Schematic diagram of RPA–CRISPR-Cas13a detection method and its process.

According to the inserted positive plasmid sequence, we used the Primer-BLAST tool of NCBI to design three sets of RPA primers, each of which was 30 nt in length, and used Weblogo^1^ to analyze the conservation of RPA primers. The location of the primers in the N sequence is shown in Table 1. The T7 promoter (20 nt) was added to the RPA upstream primer to facilitate the later transcription of the product. The crRNA design principles are provided by Changzhou Amplification Future Biotechnology Co., Ltd. Weblogo was used to analyze the conservation of the crRNA. It consists of a sequence complementary to the target gene (28 nt) and a CRISPR-related repeat sequence (36 nt), with an overall length of 64 nt. The reporter was a short RNA (Figure 1A) with a fluorescent group and quenching group added at either end, 5′ modified as FAM, 3′ modified as BHQ1, and the sequence was synthesized by Shenzhen BGI Gene Co., Ltd. The sequence designed for the experiment is shown in Table 2.

**Table 1:** Location of RPA primers in the N region of TGEV.

Primer pair	Length	Start	Stop	Product length
RPA-F1	30	177	206	306
RPA-R1	30	482	453	
RPA-F2	30	198	227	287
RPA-R2	30	484	455	
RPA-F3	30	179	208	307
RPA-R3	30	485	456	

**Table 2:** crRNA, primers (T7 promoter sequence was thickened), reporter sequence.

Name	Sequence (5′-3′)
T7-RPA-F1	**TAATACGACTCACTATAGGG**AATAGGTAACAGGGATCAACAGATTGGTTA
RPA-R1	CTTGAATTGTCTCTTGATTGATTAACTTCA
T7-RPA-F2	**TAATACGACTCACTATAGGG**GATTGGTTATTGGAATAGACAAACTCGCTA
RPA-R2	ACCTTGAATTGTCTCTTGATTGATTAACTT
T7-RPA-F3	**TAATACGACTCACTATAGGGG**TAGGTAACAGGGATCAACAGATTGGTTATT
RPA-R3	GACCTTGAATTGTCTCTTGATTGATTAACT
crRNA1	GAUUUAGACUACCCCAAAAACGAAGGGGACUAAAACUGGAUUCAUUAUUAGCACCACGACUACC
crRNA2	GAUUUAGACUACCCCAAAAACGAAGGGGACUAAAACACCUAAGUAGUAGAAGAACCACCUUUCA
reporter	6-FAM/UUUUUU/BHQ1

### 2.3 Target sequence amplification

The positive plasmid was used as the template from which to amplify the target nucleic acid sequence with a DNA rapid amplification kit (Changzhou Amplification Future Biotechnology Co., Ltd.). Buffer A (29.4 μL) was added to each reaction tube containing dry powder, and then 2 μL of primer (10 μM), 2 μL of nucleic acid template, 2.5 μL of buffer B, and 12.1 μL of water were added, mixed, and incubated at 37°C for 30 min. After the reaction, Tris-saturated phenol (25 μL), chloroform (24 μL), and isoamyl alcohol (1 μL) were added, centrifuge at 12000 rpm for 5 min at room temperature, and then extract the supernatant to obtain the purified amplification product. The RPA product was temporarily stored at −20°C.

To verify the amplification of the target sequence of RPA, 1% agarose was prepared for agarose gel electrophoresis. The first lane contained 5 μL of 100–600 bp DNA marker (Tiangen Biotechnology Co., Ltd.), and the subsequent lanes contained 6 μL of amplification product. Electrophoresis was run at 100 V. When the samples reached ¾ the length of the gel, the gel was removed and analyzed with a chemiluminescence imager.

### 2.4 CRISPR-Cas13a nucleic acid fluorescence detection

Fluorescence detection included three processes. First, the product of primer amplification was transcribed in the T7 reaction system. The T7 kit was purchased from New England Biolabs Inc. The T7 reaction system contained 8.5 μL of ddH_2_O, 1.5 μL of 10 × reaction buffer, 6 μL of NTPs (7.5 mM), 2 μL of template DNA, and 2 μL of T7 RNA polymerase (10 mM), and was incubated at 37°C for 4 h or overnight. The product was added to 1 μL of RNase inhibitor (Shanghai Beyotime Biotechnology Co., Ltd.). The nucleic acid concentration was determined using a spectrophotometer (Implen P330 NanoPhotometer), and the RNA was subsequently stored at −80°C. Cas13a protein was mixed with crRNA, after the crRNA had been diluted with diethyl pyrocarbonate (DEPC)-treated water to a final concentration of 8.0 ng/μl. The reaction system included 1 μL of 10 × reaction buffer, 2 μL of crRNA, 2 μL of Cas13a nuclease (100 ng), and 5 μL of DEPC-treated water, and incubated at 37°C for 10 min.

Finally, the CRISPR-Cas13a detection method was constructed. The reaction system included 4 μL of reaction buffer, 2 μL of ssRNA, 29 μL of DEPC-treated water, 5 μL of reporter (2 pmol/μl), and 10 μL of Cas13a–crRNA mixed solution, and was incubated at 37°C for 30 min. The results were analyzed with the Fluorescence Quantitative PCR Detection System (FQD-96A, Hangzhou Bori Technology Co., Ltd.).

### 2.5 Condition optimization

We identified the optimal reaction conditions by sequentially testing different levels of single variables and determining the final fluorescence intensity of the Cas13a-reporter. The variables included: temperature (35, 36, 37, 38, 39, and 40°C), reaction time (5, 10, 15, 20, 25, 30, 35, and 40 min), amount of RNA reporter (10, 8, 6, 4, and 2 pmol) and amount of Cas13a protein (50, 100, and 200 ng).

### 2.6 Sensitivity and specificity evaluation

In this study, we used common clinical porcine diarrhea viruses to test the specificity of the detection method, including Porcine deltacoronavirus (PDCoV), Senecavirus A (SVA), PEDV, and TGEV (The samples were from Xiao Liu laboratory, Southwest University, College of Veterinary Medicine, Chongqing, China), amplifying them with RPA and detecting them with the CRISPR-Cas13a detection method. To facilitate data collection and analysis, we used a qPCR instrument to detect samples. To evaluate the sensitivity of the detection system, we diluted the positive standard samples to a gradient (10^4^, 10^3^, 10^2^, 10^1^, and 10^0^ copies/ul) and the data results were analyzed by qPCR instrument. At the same time, were used a qPCR method to detect the same concentration gradient of these positive standard samples, and different clinical porcine diarrhea viruses, to compare the specificity and sensitivity of the two methods. The results of qPCR were analyzed with the Fluorescence Quantitative PCR Detection System and draw the standard curve.

### 2.7 Clinical sample analysis

To confirm the practicability of the established optimized detection method, clinical blood samples were acquired from pigs (Mainly 30 days piglets) in several areas of Sichuan, China. The RNA was extracted from the samples with Trizol Reagent and purified with chloroform, and isopropanol. After the reverse transcription of the sample RNA with a cDNA first-strand synthesis kit (Shanghai Biyuntian Biotechnology Co., Ltd.), the cDNA was combined with RPA–CRISPR-Cas13a. The crRNA bound to the Cas13a protein was used to identify the RNA sequence in the conserved region of the virus, and the nonspecific nucleic acid cleavage activity of Cas13a was activated. The reporter was cut to emit fluorescence, and the fluorescence was detected by visual observation under an ultraviolet light source. The Fluorescence Quantitative PCR Detection System was used to analyze the fluorescence values (Figure 1B).

## 3 Results

### 3.1 Positive plasmid verification and RPA primer screening

**Figure 2:**
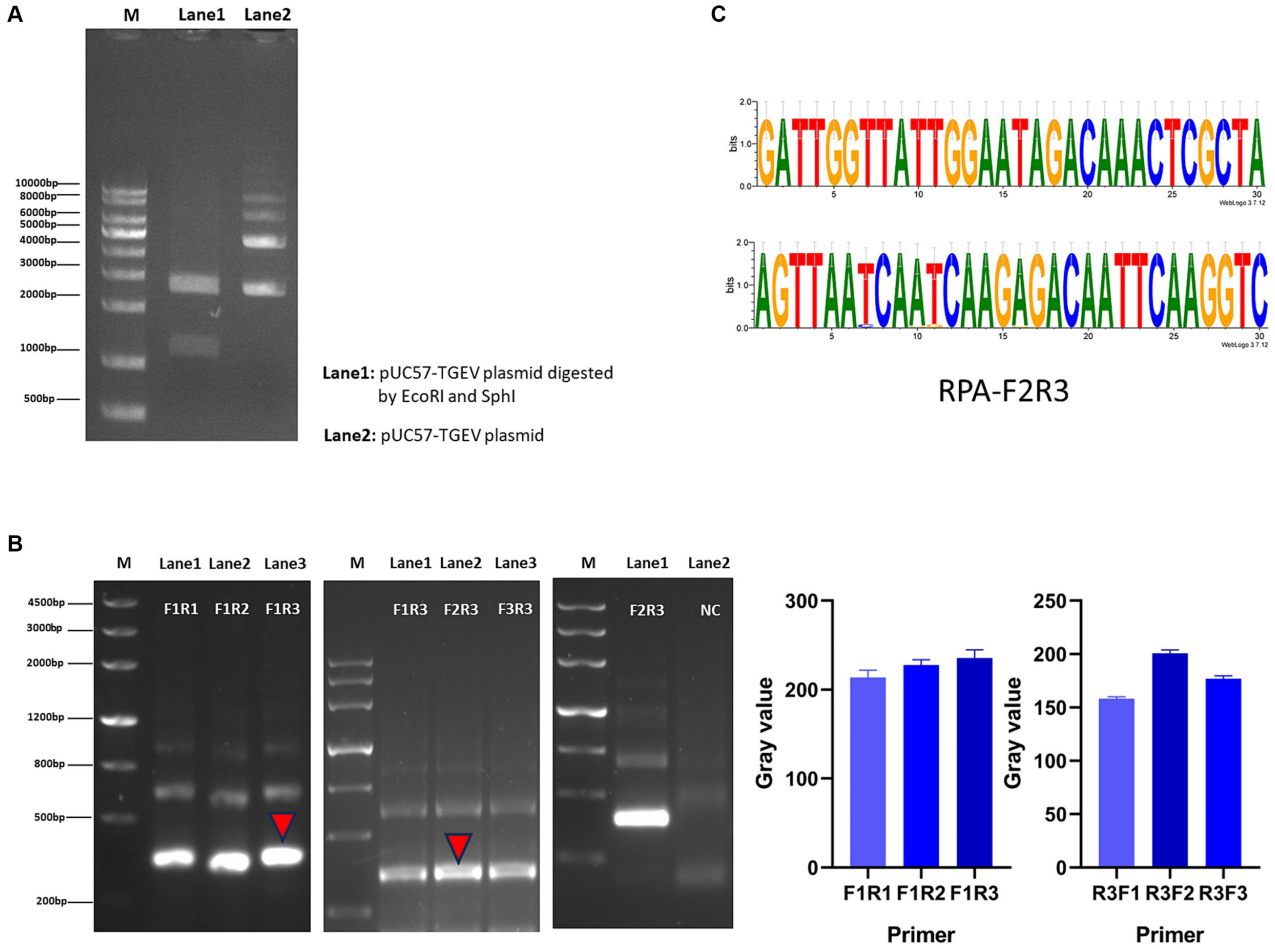
Construction of RPA–CRISPR-Cas13a detection system. **(A)** Agarose gel electrophoresis analysis of plasmid. Lane 1: double enzymatic digestion product; lane 2: the complete plasmid. **(B)** From left to right, RPA downstream primer screening, upstream primer screening, verification of the best primer pair F2R3, and the gray value of primer screening results. **(C)** Conservative analysis of the best primer pair, F2R3, with WebLogo.

We screened for transformants by culturing the cells in LB medium containing ampicillin. After the plasmid was extracted, it was doubly digested and the products resolved with agarose gel electrophoresis. Two bands of about 1,100 bp and 2,700 bp were detected, confirming that the positive plasmid was successfully constructed (Figure 2A). Using the positive plasmid as the DNA template, three pairs of RPA primers were tested for RPA amplification. After agarose gel electrophoresis, images were obtained with a chemical imager, and analyzed with the ImageJ software. Finally, the F2R3 primers pair was identified as providing the highest amplification efficiency (Figure 2B). After conducting analysis using WebLogo, it was confirmed that the F2R3 primers exhibit high conservatism (Figure 2C).

### 3.2 Optimization of RPA–CRISPR-Cas13a fluorescence detection conditions

**Figure 3:**
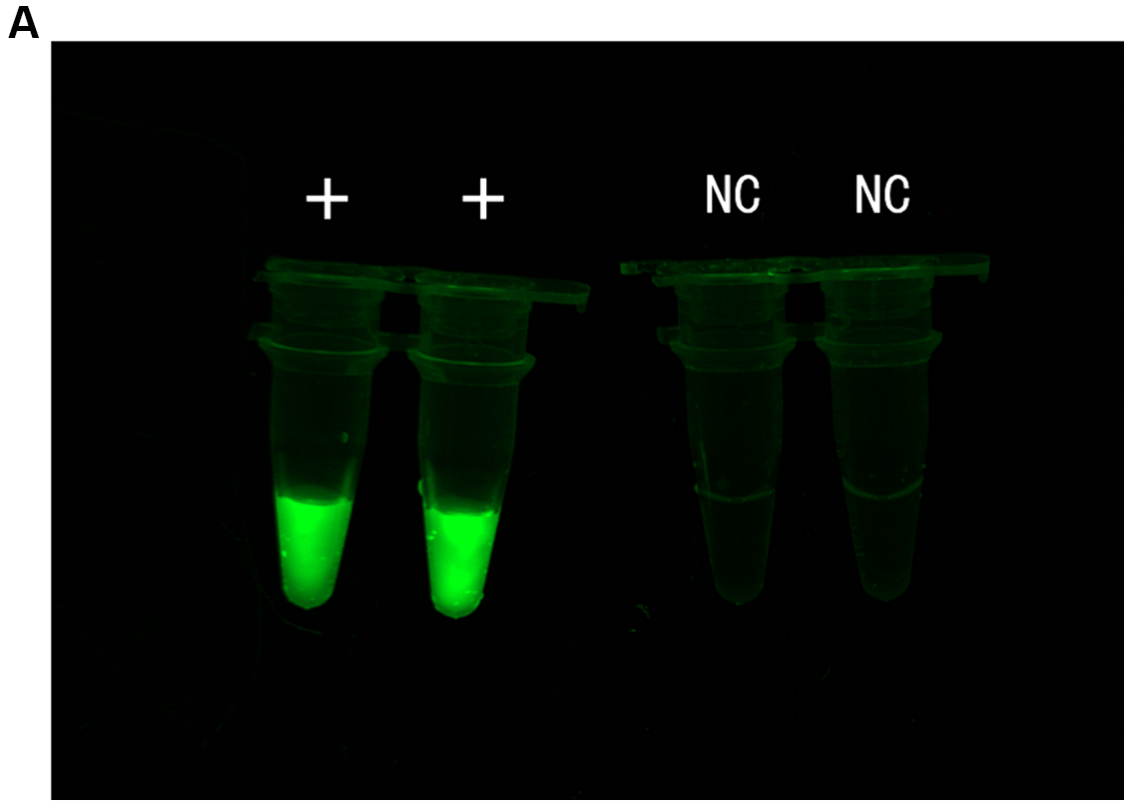
Verify RPA–CRISPR-Cas13a detection system. **(A)** From left to right, positive control group, negative control group.

**Figure 4:**
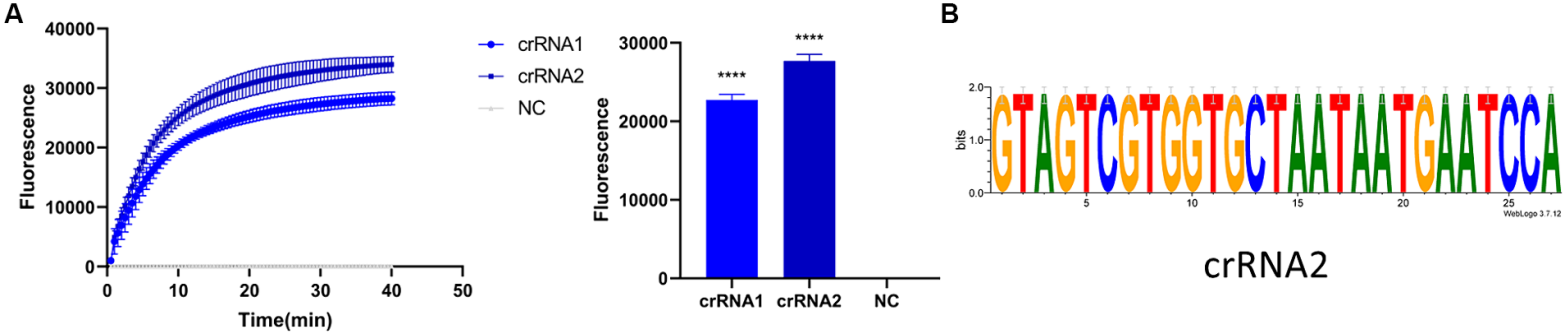
Screening of crRNA. **(A)** Detection efficiency of crRNA2 was significantly higher than that of crRNA1. **(B)** Verifying the conservativeness of crRNA2 with WebLogo (****p < 0.0001).

**Figure 5:**
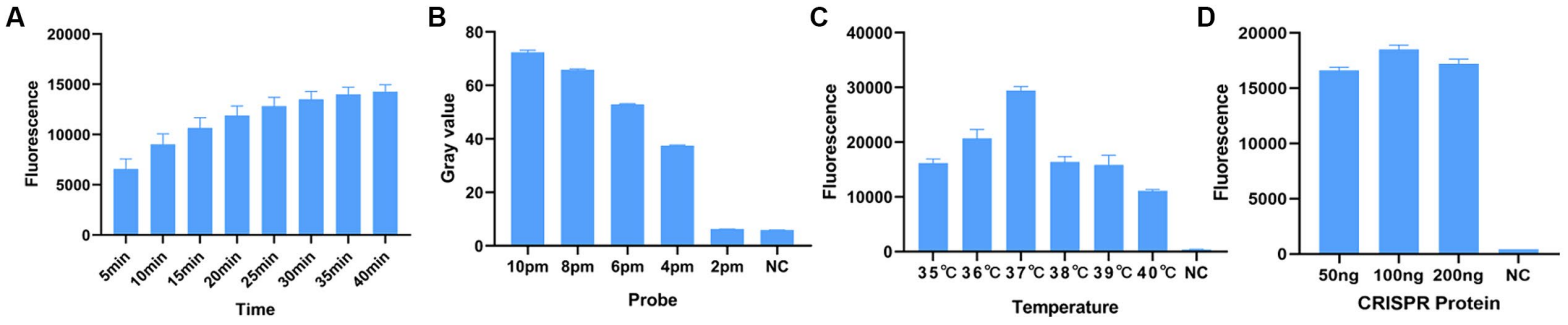
Optimization of RPA–CRISPR-Cas13a reaction conditions. **(A)** Optimal reaction time. When the reaction proceeded for about 30 min, the fluorescence intensity did not differ greatly from other reaction times, so to minimize the reaction time, 30 min was selected. **(B)** Optimal amount of probe. The fluorescence of the reaction was obvious when the probe concentration was 10 pmol, 8 pmol, and 6 pmol. To minimize the amount of probe used, 6 pmol was selected as the optimal amount of probe. **(C)** Optimal reaction temperature. Fluorescence intensity was greatest when the reaction temperature was 37°C. **(D)** Optimal amount of Cas13a protein. Fluorescence intensity was greatest when the amount of protein used was 100 ng.

Before optimizing the conditions, we verified the RPA-CRISPR-Cas13a reaction system. The results showed that the positive control group emitted fluorescence under blue-violet light, and the negative control did not emit fluorescence (Figure 3A). Then, we optimized the crRNA, reaction time, temperature, amount of reporter, and amount of Cas13a protein. RPA amplification was performed with the F2R3 primers. After T7 transcription combined with CRISPR–Cas13a, the results were analyzed with qPCR instrument to identify the optimal crRNA, crRNA2 (Figure 4A). WebLogo analysis was used to compare multiple strains, and the results showed that the crRNA sequence was highly conserved (Figure 4B). The fluorescence intensity of the reaction system was high and the change in fluorescence was small when the reaction was performed for about 30 min, so 30 min was selected as the best reaction time (Figure 5A). When used different amount of reporter, the results of 10, 8, and 6 pmol showed high fluorescence intensity. In order to save consumables, so 6 pmol was considered the optimal amount of reporter (Figure 5B; Supplementary Figure S1). The optimized temperature and amount of Cas13a were 37°C (Figure 5C) and 100 ng (Figure 5D), respectively.

### 3.3 RPA–CRISPR-Cas13a detection ability

**Figure 6:**
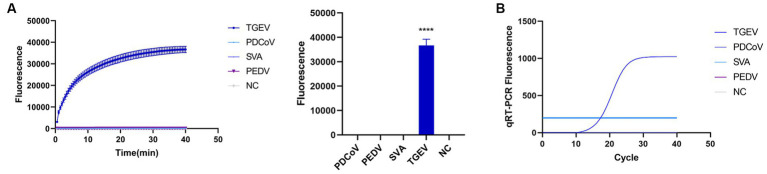
Verifying the specificity of the detection system. **(A)** Used the established method to detect TGEV, PDCoV, SVA and PEDV. Only the reaction containing TGEV emitted fluorescence. **(B)** specificity of qPCR detection method (****p < 0.0001).

**Figure 7:**
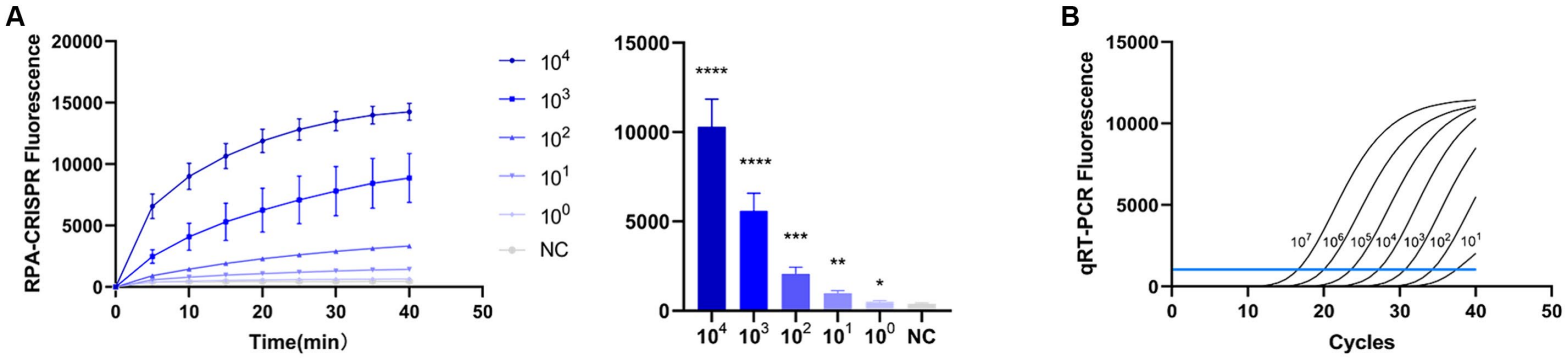
Sensitivity of RPA–CRISPR-Cas13a is consistent with that of qPCR. **(A)** Sensitivity of RPA–CRISPR-Cas13a detection method. The system detected 10^1^ copies of TGEV. **(B)** Sensitivity of qPCR detection method. (****p < 0.0001, ***p < 0.001, **p < 0.01, *p < 0.1).

To evaluate the detection capacity of the CRISPR-Cas13a-associated detection method, we tested its specificity for TGEV, SVA, PDCoV, and PEDV. The detection result showed that only the TGEV group tested positive (Figure 6A), which is consistent with the qPCR method (Figure 6B). This indicates that the CRISPR-Cas13a-associated detection method exhibits good specificity. Repeated tests confirmed the specificity of the assay for TGEV to be 100%. We then verified the sensitivity of the detection system and compared it with the qPCR method. Gradient dilutions of the positive samples were used as the detection templates. Since the test results were analyzed based on the final fluorescence signal intensity, a fluorescence intensity higher than 1447.56 was considered indicative of a positive result. The results showed that the established CRISPR-Cas13a-associated detection method generated a fluorescent signal at a minimum of 7.7 × 10^1^ copies of the virus (Figure 7A). Additionally, a qPCR method was employed to detect the same concentration gradient of these samples, with all samples exhibiting Ct values ≤35 being classified as positive. The qPCR showed a sensitivity of 7.7 × 10^2^ copies (Figure 7B), confirming that our detection method is highly sensitive.

To test the repeatability of the assay, we divided the same sample into three concentrations (10^5^, 10^4^, and 10^3^ copies/ul), and repeated the test in 3 days. The coefficients of variation were 1.06, 4.13, and 16.65% (Supplementary Table S1), respectively. The negative control group emitted no fluorescence. These results indicate that the repeatability of the assay was good.

### 3.4 Detection of clinical samples in areas of Sichuan, China

Using the established detection system, we tested field samples from different farms, including Mingxing (MX), KA, and Yuanda (YD), in different regions of Chengdu, Leshan, and Guangyuan (Sichuan Province, China). We randomly selected 123 samples from the entire collection and utilized the CRISPR-Cas13a-assisted detection method to analyze these samples. The results of the CRISPR–Cas13a-assisted detection system were compared with the results of qPCR. The sensitivity, specificity, positive coincidence rate, negative coincidence rate, and kappa value of the CRISPR–Cas13a-assisted detection method were 100, 98.93, 96.66, 100%, and 0.97 (Table 3), respectively. These results were very close to the results of qPCR, confirming that the CRISPR–Cas13a-assisted detection system is a very reliable detection technology.

**Table 3:** qPCR as the gold standard method.

	qPCR			Sensitivity	Specificity	PPA	NPA	Kappa value
		Positive	Negative					
CRISPR-fluorescence	Positive	29	1	100%	98.93%	96.66%	100%	0.97
	Negative	0	93					
	Total	29	94					

Evaluation of the authenticity and reliability of RPA–CRISPR-Cas13a.

## 4 Discussion

Since TGEV was discovered in the United States in 1946 (15), it has spread all over the world, bringing huge economic losses to the world pig-breeding industry. The prevalence of TGEV in China is not particularly serious (16), and the positivity rate in pigs is low. However, it is noteworthy that China is a large pig-breeding country. As an RNA virus, TGEV is highly mutable. For example, the emergence of its respiratory mutant, porcine respiratory coronavirus (PRCV), demonstrates the risk it potentially poses. TGEV has a 100% mortality rate in piglets. Consequently, a mutation of the virus or an unpredictable sudden outbreak of a TGEV epidemic increases the pressure on China’s pig-breeding industry to introduce prevention and control measures. When piglets are infected with TGEV, they are likely to die, and there is as yet no drug treatment. Most pigs have poor immune effect after vaccination (4, 5), so the timely diagnosis of TGEV infection is extremely important.

At present, the laboratory diagnosis of TGEV is mostly using the traditional PCR amplification technology, which is strongly dependent upon instrumentation, and this requirement cannot be met in many regions of developing countries. In 2006, Piepenburg et al. developed an RPA technology to replace PCR, and this reaction is both sensitive and fast. Importantly, the reaction is performed at a constant low temperature, and it does not require the cumbersome cycling steps of the PCR technology (17). Compared with PCR technology, the emergence of the RPA technology provides a new opportunity for real-time and on-site detection scenarios (18). The RPA technology reduces the reliance on instruments and diminishes the need for strict temperature control. This technology helps to streamline processes and reduce the dependency on precise environmental conditions (17). Therefore, the RPA technology is receiving increasing attention, with continuing innovations and improvements, including RPA combined with lateral flow strips, multiplex RPA, RPA-related doubleplex isothermal molecular detection method, etc. (19–21). RPA is also being used in the detection of bacteria, fungi, viruses, parasites, genetically modified foods, and the genes of drug-resistant bacteria (22–24). Its function has been affirmed in various fields.

CRISPR-Cas is an adaptive immune system of prokaryotes. Cas proteins recognize and cut foreign nucleic acid sequences and store them in CRISPR spacer regions (12). With the in-depth study of the CRISPR system, the discovery of Cas9 led to the discovery of its potential utility in DNA editing (25). The characterization of the Cas13 protein identified its RNA splicing characteristics, which are now used in RNA-related gene editing. Moreover, using the nuclease activity of Cas13 protein, researchers have innovatively added reporter RNAs with a FAM fluorescent group to the system, and constructed a diagnostic method for nucleic acids *in vitro* (26–28). In 2017, the Zhang Feng laboratory constructed a nucleic acid detection method based on CRISPR-Cas13a (27). Recently, this technology has been used in the detection of viruses.

Given the shortcomings of the current clinical diagnostic technology for TGEV, we established a novel detection method for TGEV based on RPA and CRISPR-Cas13a. Its requirements for equipment are low. Compared with qPCR, it only requires a constant temperature heating device and a blue-violet flash to complete the detection of the sample, and it can complete the detection of TGEV at a low temperature and the interpretation of the results is relatively simple. As a proven excellent detection method, qPCR is widely used (29). Therefore, we compared the CRISPR–Cas13a-assisted detection method with the qPCR detection method to evaluate the sensitivity and specificity of the CRISPR–Cas13a-assisted detection method. In the study, only TGEV showed the positive results in both qPCR and CRISPR-Cas13a-assisted detection method. In the sensitivity test, the detection limits of the two methods were 10^1^ copies and 10^2^ copies, respectively. In this study, qPCR was only used as a reference detection method, the reaction system and primers were not optimized, results of qPCR may be affected by the primers, but it can still reflect its specificity and sensitivity. Then we verified the repeatability of the established RPA-CRISPR-Cas13a detection method. The coefficient of variation of the samples at 10^3^ copies was higher than 10^5^ and 10^4^ copies. It is possible that the low-copies samples more susceptible to the effects of long-term storage and repeated freeze–thaw cycles, which could lead to greater variability in results. We optimized several of the reaction conditions, including the reaction time, amount of Cas13a protein used, and amount of reporter used, to make it more practicable and economical within a certain range and to avoid the waste of reagents. The RPA–CRISPR-Cas13a-assisted detection method provides a more convenient and highly sensitive option for the detection of TGEV in developing countries, in small enterprises, or on private farms. Although the detection system still has some drawbacks, such as the need to avoid sample contamination, inability to completely eliminate dependence on instruments, high RPA costs, and multiple operational steps, but we believe that the defects of this new detection method do not obscure its virtues. During the experiment, we noted that the CRISPR-Cas13a, and T7 transcription, can be performed at 37°C, so these steps can be integrated into one step. In addition, we can try to integrate most of the reagents into a reaction system. When used on site, it can reduce the operation steps and training requirements for personnel, and save time. In summary, the whole detection method shows good specificity, sensitivity, and repeatability and will facilitate the detection of TGEV and assist in the prevention and control of TGEV-associated disease.

## Data availability statement

Publicly available datasets were analyzed in this study. This data can be found at: https://www.ncbi.nlm.nih.gov/nuccore/2428737012.

## Ethics statement

The animal study was approved by Laboratory Animal Center, Southwest University. The study was conducted in accordance with the local legislation and institutional requirements.

## Author contributions

HW: Writing – original draft, Data curation, Software, Methodology. ZC: Writing – original draft. LR: Writing – original draft. QY: Writing – original draft. YZ: Writing – original draft. YY: Writing – original draft. YC: Writing – original draft. WL: Funding acquisition, Writing – review & editing. XL: Funding acquisition, Methodology, Writing – review & editing.

## Footnotes

^1^ https://weblogo.threeplusone.com
