# Supplementary material for: RPA-CRISPR-Cas13a-assisted detection method of transmissible gastroenteritis virus
Source: Front Vet Sci. 2024 Jul 2;11:1428591. doi: 10.3389/fvets.2024.1428591 (PMC11249537; doi:10.3389/fvets.2024.1428591)
Supplement: Supplementary file 1 [file Data_Sheet_1.DOCX]

Supplementary Material

# Supplementary Figures and Tables

## Supplementary Figures

## Supplementary Figure S1. (A). The detection results of TGEV of RPA-CRISPR-Cas13a under different concentrations of probes under blue-violet light.
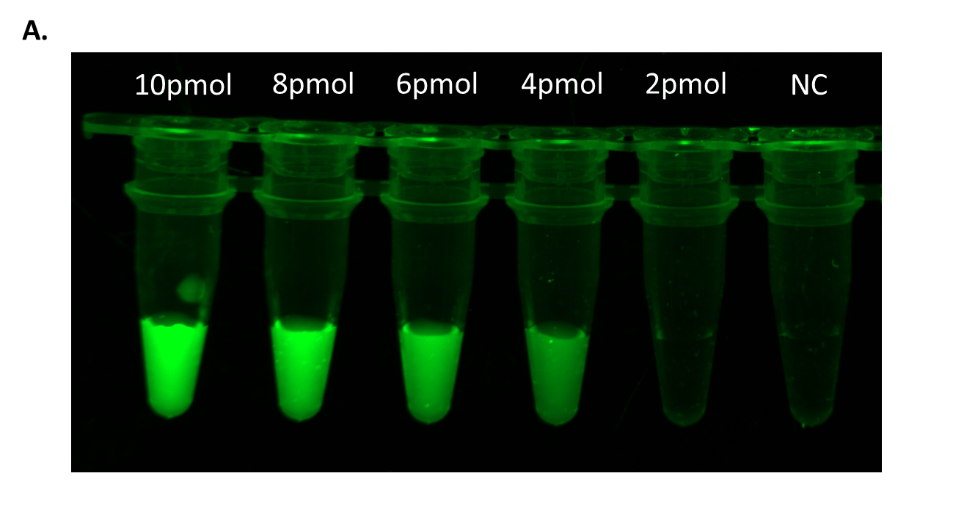


## Supplementary Tables

## Supplementary Table S1. Repeatability verification.

|  | Sample | | |
| --- | --- | --- | --- |
| (copies/ul) | 10^5^ | 10^4^ | 10^3^ |
| Coefficient of Variation | 1.06% | 4.13% | 16.65% |
